# The combination of dehydroepiandrosterone, transdermal testosterone, and growth hormone as an adjuvant therapy in assisted reproductive technology cycles in patients aged below 40 years with diminished ovarian reserve

**DOI:** 10.4274/tjod.32656

**Published:** 2015-06-15

**Authors:** Bülent Haydardedeoğlu, Ahmet Zeki Işık, Esra Bulgan Kılıçdağ

**Affiliations:** 1 Başkent University Faculty of Medicine, Department of Obstetrics and Gynecology, Adana, Turkey; 2 İzmir University Faculty of Medicine, Department of Obstetrics and Gynecology, İzmir, Turkey

**Keywords:** Diminished ovarian reserve, DHEA, transdermal testosterone, growth hormone, IVF/ICSI

## Abstract

**Objective:**

To evaluate to the efficacy of testosterone, dehydroepiandrosterone (DHEA) and growth hormone (GH) supplementations in patients with diminished ovarian reserve (DOR) in assisted reproductive technology (ART) cycles.

**Materials and Methods:**

A retrospective cohort including 33 women with 81 ART cycles were aged and ovarian reserve matched 52 women with 102 conventional in vitro fertilization (IVF)/intra-cytoplasmic sperm injection (ICSI) protocol. Administration of DHEA for 12 weeks and transdermal testosterone for 4 weeks as pretreatment adjuvant and luteal start GH in DOR patient treatment arm compared to conventional IVF/ICSI cycles.

**Results:**

The number of follicles >14 mm, number of oocytes, number of metaphase 2 oocytes and fertilisation rate were significantly higher in ISIK protocol (IP). The clinical pregnancy rate (CPR) per embryo transfer of the IP was 38.2% (13/34). The cancellation rate of cycles decreased significantly from 54.5 % (24/44) to 8.1% (3/37) with the IP, while the OPR was 35.3% (12/34).

**Conclusions:**

Our study has shown that even the poorest responders could achieve clinical pregnancy after inducing ovarian folliculogenesis with a combination of transdermal testosterone, DHEA and GH.

## INTRODUCTION

Diminished ovarian reserve (DOR) is one of the most challenging conditions for an assisted reproductive technology (ART) specialist due to the high cancellation rate and low live birth rate (LBR). Although many controlled ovarian hyperstimulation (COH) protocols have been proposed for patients with DOR, similar LBRs have been observed^(1)^. ART cycle outcomes in DOR patients have been claimed to be improved by pre- and co-treatment with several drugs as adjuvants to COH, such as administration of transdermal testosterone, dehydroepiandrosterone (DHEA), and growth hormone (GH)^(2,3,4)^.

Androgens have an important role in ovarian folliculogenesis, and a decrease in clinical pregnancy rate in patients undergoing IVF has been shown to be related to lower testosterone levels^(5)^. Gleicher et al. demonstrated significant hypoandrogenism in women with DOR compared with age-matched women with normal ovarian reserve^(6)^. Although there is no strong consensus on the positive effect of DHEA supplementation for patients with DOR^(7)^, recent literature favors the administration of DHEA^(8,9,10)^.

The efficacy of testosterone, DHEA, and GH supplementation has been studied in patients with DOR separately; however, the combination of these alternative regimens has not yet been reported. This study evaluated the outcome of in vitro fertilization (IVF)/intra-cytoplasmic sperm injection (ICSI) cycles. Before the commencement of COH, we combined a 12-week DHEA administration with the addition of transdermal testosterone for the last 4 weeks and GH supplementation initiated in the late luteal phase in patients with DOR who had previously cancelled or failed IVF/ICSI cycles.

## MATERIALS AND METHODS

This study was conducted in Başkent University Faculty of Medicine, Department of Obstetrics and Gynecology, Division of Reproductive Endocrinology and IVF Unit (Adana, Turkey), and approved by the Ethics Committee of Başkent University. We retrospectively enrolled 81 IVF/ICSI cycles in 33 patients from October 2010 to December 2013. We compared 44 cycles in 33 women who had resulted in cancellation or pregnancy failure with 37 cycles during which a novel treatment protocol was used, which we called the ISIK protocol (IP). Before the start of COH, 12 weeks of DHEA 25 mg t.i.d in combination with 25 mg transdermal testosterone gel daily for the last 4 weeks, and 3 IU GH administration was started in the late luteal phase.

We also defined a control group through the retrospective analysis of 2155 IVF/ICSI cycles that were performed during the same time period. In total, 51 patients were found to be appropriate for comparing with the study group in terms of age and ovarian reserve parameters. The patients in the control group had undergone 102 conventional IVF/ICSI cycles in our clinic during this time period.

The diagnosis of DOR was made in accordance with the European Society of Human Reproduction and Embryology (ESHRE) Consensus of Bologna criteria for the study cohort and control group^(11)^. The previous 44 cycles of the 33 patients consisted of 24 flexible gonadotropin releasing hormone (GnRH) antagonist cycles with 300 IU of recombinant follicle-stimulating hormone (FSH), 20 cycles of clomiphene citrate, and low-dose (150 IU) recombinant FSH combined with a GnRH antagonist. These patients were scheduled for IP, which was initiated by the administration of 75 mg/d DHEA (Biosterone 25 mg, Interpharm, Switzerland) for 8 weeks. Throughout DHEA administration, antral follicle count was examined upon each menstruation. Thereafter, 25 mg/d transdermal testosterone gel (Testogel 50 mg, Bayer Pharma AG, Berlin, Germany), 75 mg/d DHEA, and an oral contraceptive pill (Yasmin, Bayer Pharma AG, Berlin, Germany) for 21 days starting on the first day of menstruation. Growth hormone was started as 1 mg/3 IU (Genotropin, Pfizer Inc., New York, NY, USA) on day 21 of the last menstrual cycle. Subcutaneous injection of GH was applied every other day (q.o.d.) till the start of menstruation. DHEA and testosterone gel application were stopped on the first day of the menstrual cycle.

**Figure 1:**
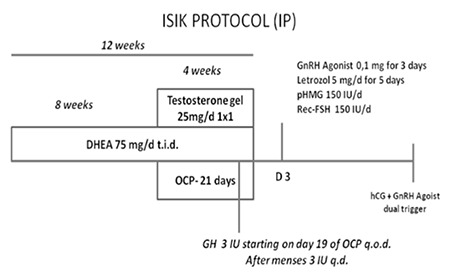
ISIK protocol schematic view

On the third day of the menstrual cycle, GH administration was shifted to daily 3 IU injections and in addition to this, 0.1 mg/ml of the GnRH agonist triptoreline (Decapeptyl; Ferring Pharmaceuticals, Switzerland) for 3 days and 5 mg/d letrozol (Femara; Novartis Pharma AG, Basel, Switzerland) for 5 days were commenced. Administration of gonadotropins was also started on the same day with 150 IU of recombinant FSH (Puregon; MSD, The Netherlands), plus 150 IU of pure human menopausal gonadotropin (HMG) (Menopur; Ferring Pharmaceuticals, Switzerland). A GnRH antagonist (Orgalutran; MSD, The Netherlands) was added to this regimen when the leading follicle reached 14 mm (Figure 1).

After the leading follicle reached >17 mm, 10.000 IU of hCG (Pregnyl ampul; MSD, The Netherlands) and 0.2 mg/ml triptorelin were injected. Transvaginal ultrasound-guided oocyte retrieval was performed 36 h after this dual trigger with a 17-gauge needle under sedation with 1% propofol (Fresenius Kabi, Germany). The oocyte-corona complexes (OOC) were denuded, and ICSI was performed after 2-h incubation. Embryos were transferred on day 3.

All patients had luteal support with intravaginal daily 90-mg progesterone (Crinone 8% gel, Merck Serono) and 0.1 mg/ml triptorelin on the third day after embryo transfer^(12)^. Clinical pregnancy was defined as the presence of at least one gestational sac with detectable fetal cardiac activity under transvaginal ultrasonography.

### Statistical Analysis

Data are expressed as means ± SD. The baseline differences among the previous and study cycles were analyzed by one-way analysis of variance. A Bonferroni test was used for post hoc comparisons. In contingency tables, the χ^2^ test or the two-sided Fisher’s exact test was performed. P<0.05 was considered to indicate statistical significance. Data were analyzed using SPSS for Windows (version 16.0; SPSS, Inc., Chicago, IL).

## RESULTS

Baseline characteristics of the two protocols are shown in Table 1. Although baseline characteristics were similar, the anti-Müllerian hormone (AMH) level of the control group was higher. The duration of COH cycles was significantly longer in the ISIK protocol. However, the number of follicles >14 mm, number of oocytes, number of metaphase 2 oocytes and fertilization rate were significantly higher in IP (Table 1). Although the fertilization rate of the control group was lower than IP, the difference was not statistically significant. The fertilization rate of the study group was statistically lower than IP (Table 1). The mean number of grade 1 and transferred embryos were similar in both groups (Table 1). In the IP group, four patients had 2 IVF/ICSI cycles (33 patients had 37 cycles and 34 transfers). Three cycles were repeated due to fertilization failure. The fourth patient had a second IP cycle due to the negative result of the first cycle.

The clinical pregnancy rate per embryo transfer of IP was 38.2% (13/34) (Table 2). The cancellation rate of cycles significantly decreased from 54.5% (24/44) to 8.1% (3/37) with IP, and the ongoing pregnancy rate was 35.3% (12/34 embryo transfer). However, clinical pregnancy rate (CPR) and LBR in the control group were significantly lower than IP (22% and 10%, respectively). Although the implantation rate (IR) was lower in the control group than IP, the difference did not reach statistical significance.

## DISCUSSION

This protocol was developed in two clinics based on long discussions and the accumulation of data supporting the use of DHEA and transdermal testosterone in patients with DOR. Unexpected and astonishing oocyte yields and deliveries in patients with very high FSH levels, ranging from 25-89 IU/L during a relatively short period of time (unpublished data) led us to attempt this protocol in a well-defined group of poor-responder patients. We analyzed the data retrospectively in such a patient cohort from whom all records were available for the previous cancelled or failed cycles.

Our study has shown that even the poorest responders could achieve clinical pregnancy after inducing ovarian folliculogenesis with a combination of transdermal testosterone, DHEA, and GH.

Although short-term DHEA supplementation in DOR does not increase AMH levels, an increase in antral follicle count after 16 weeks of supplementation has been reported recently^(13)^. Moreover, DHEA supplementation has been associated with unexpected spontaneous pregnancies prior to IVF^(10)^. This effect of DHEA may be explained by the finding of reduction in the embryonic aneuploidy rate^(14)^. In an animal model, 10 weeks of DHEA supplementation in 6 female sheep resulted in increased expression of the proliferation marker Ki-67 in granulosa cells and follicular AMH expression in the pre-antral and early antral follicle stages^(15)^. Surprisingly, a recent but previous meta-analysis of human studies by the same authors demonstrated that DHEA supplementation in DOR had no significant effect on oocyte yield and CPR^(16)^. There was a reduction in the androgenic milieu during the late follicular stage in patients with DOR, and de los Santos et al. suggested that long-term androgen supplementation for oocyte priming could increase recruitment of small antral follicles rather than increase intra-ovarian androgen levels in women with DOR^(17)^.

The role of androgens in folliculogenesis remains a matter of debate. Although short-term administration of high doses of androgens in monkeys did not increase the number of large antral follicles, the total number of growing pre-antral and small antral follicles was increased 2.5-4.5- fold^(18)^. The first randomized controlled trial (RCT) on testosterone pre-treatment in women with DOR demonstrated that even though IVF/ICSI cycle outcomes were similar, testosterone pre-treatment improved ovarian sensitivity and follicular response to gonadotropins^(19)^. Another RCT of transdermal testosterone pre-treatment (TTP) demonstrated significantly improved cycle outcomes^(20)^. These two RCTs did not comment on the dermatologic adverse effects of androgen supplementation and there have been no reports of DHEA and transdermal testosterone combination therapy. Our retrospective cohort is the first clinical study of this combined approach. The cycle outcomes in our study group support the results of other clinical trials in which DHEA and TTP were used as adjuvants. Moreover, a meta-analysis by Bosdou et al. demonstrated better cycle outcomes when androgens or androgen-modulating agents were used in patients with patients DOR^(21)^.

The current study demonstrated that total gonadotropin use was relatively low with the conventional protocol. This was related to shorter COH duration and the administration of 20 cycles of clomiphene citrate. The shorter COH duration could be related to higher basal FSH levels in the poorest responders, which might lead to earlier dominant follicle development that impaired follicular synchronization in the COH cycle. Rapid follicular development could also lead to premature luteinization and the addition of GnRH antagonist, which is proposed to prevent this, may also suppress the growth of the aftercoming follicular cohort. Pre-treatment with DHEA and testosterone together with GH might increase intra-ovarian androgen levels, resulting in slower follicle growth with IP, which could enhance the development of a few more antral follicles and allow for a more homogenous maturation. For the time being, we do not know which agent or agents mediate this response.

In addition to the meta-analysis by Duffy et al., another meta-analysis of GH co-treatment in COH cycles in DOR also demonstrated that the addition of GH improved the rate of clinical pregnancy and LBR^(4,22)^. It has been shown that oral DHEA supplementation increased serum insulin-like growth factor-I concentrations, which positively affected follicular development and oocyte quality^(23)^; GH supplementation in DOR may function in a similar manner. Our study also demonstrated that the combination of DHEA and GH might have additive effects that cause higher oocyte numbers and better cycle outcomes in DOR patients. On the other hand, our COH cycle management was different from other published protocols. We administered a GnRH analogue for 3 days together with recombinant FSH plus HMG, a total of 300 IU/d, and added 5 mg/d letrazole for 5 days. The aim of the GnRH agonist ultra-short application was to induce endogenous FSH together with exogenous gonadotropins. Letrozole was added to induce a similar effect and to reduce the peripheral estrogen, which might be a preventive measure against a premature LH surge. Also, a relatively low estrogenic milieu resembling physiologic levels may lead to better endometrial growth for improved implantation and cycle outcomes. We stopped administration of DHEA and testosterone throughout the COH cycle due to potential adverse effects of excess androgens on the endometrium. Weissman et al. reported increased follicular phase progesterone levels in COH cycles during which DHEA was co-administered^(24)^. DHEA was very recently shown to induce a progesterone rise that resolves rapidly after cessation of the drug, which may be detrimental to the endometrium^(25)^.

Oocyte donation is not permitted in Turkey; thus, IP was developed as a last resort in our cohort so as to perform IVF/ICSI with patients’ own gametes. The unexpected higher pregnancy rates could be attributed to the enrolment of relatively younger women with DOR. Another interesting finding was the higher fertilization rate in the IP group, which could be explained by improved oocyte quality due to the addition of GH and DHEA supplementation, which reduced embryo aneuploidy in a previous study^(14)^. Although the embryo qualities of both groups were similar, the increased fertilization rate appeared to be a function of both DHEA and GH.

A major limitation of this study is that it was a retrospective cohort using the same group of women with DOR. In addition, the combination of effective treatment modalities may mask the independent effects of individual medications. It should be noted that patients with DOR use a variety of nutritional and herbal supplements, and continue their use even with conventional controlled ovarian stimulation protocols. In addition, attempting an RCT is difficult in patients DOR because most patients want to be in the study group and will not give informed consent. We tried to establish a novel pre- and co-treatment modality using drugs that are individually effective on a physiologic basis, which when combined may have had synergistic effects on oocytes, embryos, and possibly on endometrial quality. The combination of the androgens DHEA, and TTP with GH should be evaluated in large-scale, well-designed, randomized trials that involve both young and advanced-age patients with DOR.

## Figures and Tables

**Table 1:**
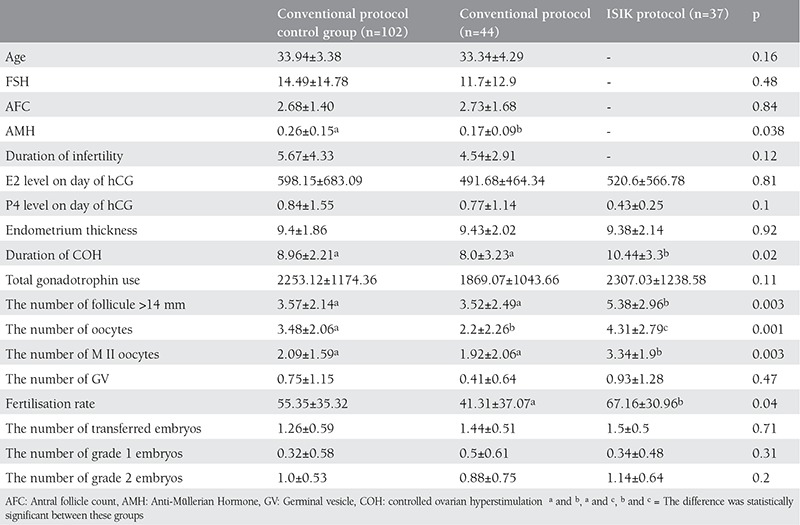
Basal characteristics of study groups

**Table 2:**
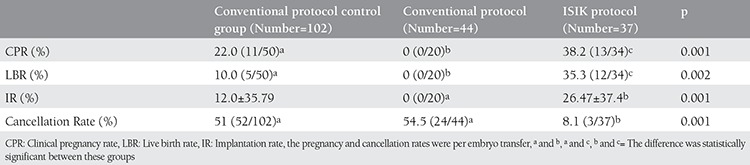
IVF/ICSI outcomes of study groups

## References

[1] Pandian Z, McTavish AR, Aucott L, Hamilton MP, Bhattacharya S (2010). Interventions for ‘poor responders’ to controlled ovarian hyper stimulation (COH) in in-vitro fertilisation (IVF). Cochrane Database Syst Rev.

[2] Sunkara SK, Pundir J, Khalaf Y (2011). Effect of androgen supplementation or modulation on ovarian stimulation outcome in poor responders: a meta-analysis. Reprod Biomed Online.

[3] González-Comadran M, Durán M, Solà I, Fábregues F, Carreras R, Checa MA (2012). Effects of transdermal testosterone in poor responders undergoing IVF: systematic review and meta-analysis. Reprod Biomed Online.

[4] Duffy JM, Ahmad G, Mohiyiddeen LG, Nardo LG, Watson A (2010). Growth hormone for in vitro fertilization. Cochrane Database Syst Rev.

[5] Lu Q, Shen H, Li Y, Zhang C, Wang C, Chen X, Liang R, Wei L (2014). Low testosterone levels in women with diminished ovarian reserve impair embryo implantation rate: a retrospective case-control study. J Assist Reprod Genet.

[6] Gleicher N, Kim A, Weghofer A, Kushnir VA, Shohat-Tal A, Lazzaroni E, Lee HJ, Barad DH (2013). Hypoandrogenism in association with diminished functional ovarian reserve. Hum Reprod.

[7] Yakin K, Urman B (2011). DHEA as a miracle drug in the treatment of poor responders; hype or hope?. Hum Reprod.

[8] Wiser A, Gonen O, Ghetler Y, Shavit T, Berkovitz A, Shulman A (2010). Addition of dehydroepiandrosterone (DHEA) for poor-responder patients before and during IVF treatment improves the pregnancy rate: a randomized prospective study. Hum Reprod.

[9] Gleicher N, Barad DH (2012). Hype or hope? Ethical and practical considerations with clinical research in women with diminished ovarian reserve. Reprod Biomed Online.

[10] Fusi FM, Ferrario M, Bosisio C, Arnoldi M, Zanga L (2013). DHEA supplementation positively affects spontaneous pregnancies in women with diminished ovarian function. Gynecol Endocrinol.

[11] Ferraretti AP, La Marca A, Fauser BC, Tarlatzis B, Nargund G, Gianaroli L, ESHRE working group on Poor Ovarian Response Definition (2011). ESHRE consensus on the definition of ‘poor response’ to ovarian stimulation for in vitro fertilization: the Bologna criteria. Hum Reprod.

[12] Isik AZ, Caglar GS, Sozen E, Akarsu C, Tuncay G, Ozbicer T, Vicdan K (2009). Single-dose GnRH agonist administration in the luteal phase of GnRH antagonist cycles: a prospective randomized study. Reprod Biomed Online.

[13] Yeung TW, Li RH, Lee VC, Ho PC, Ng EH (2013). A randomized double-blinded placebo-controlled trial on the effect of dehydroepiandrosterone for 16 weeks on ovarian response markers in women with primary ovarian insufficiency. J Clin Endocrinol Metab.

[14] Gleicher N, Weghofer A, Barad DH (2010). Dehydroepiandrosterone (DHEA) reduces embryo aneuploidy: direct evidence from preimplantation genetic screening (PGS). Reprod Biol Endocrinol.

[15] Narkwichean A, Jayaprakasan K, Maalouf WE, Hernandez-Medrano JH, Pincott-Allen C, Campbell BK (2014). Effects of dehydroepiandrosterone on in vivo ovine follicular development. Hum Reprod.

[16] Narkwichean A, Maalouf W, Campbell BK, Jayaprakasan K (2013). Efficacy of dehydroepiandrosterone to improve ovarian response in women with diminished ovarian reserve: a meta-analysis. Reprod Biol Endocrinol.

[17] Santos MJ, García-Laez V, Beltrán D, Labarta E, Zuzuarregui JL, Alamá P, Gámiz P, Crespo J, Bosch E, Pellicer A (2013). The follicular hormonal profile in low-responder patients undergoing unstimulated cycles: Is it hypoandrogenic?. Hum Reprod.

[18] Vendola KA, Zhou J, Adesanya OO, Weil SJ, Bondy CA (1998). Androgens stimulate early stages of follicular growth in the primate ovary. J Clin Invest.

[19] Fábregues F, Peñarrubia J, Creus M, Manau D, Casals G, Carmona F, Balasch J (2009). Transdermal testosterone may improve ovarian response to gonadotrophins in low-responder IVF patients: a randomized, clinical trial. Hum Reprod.

[20] Kim CH, Howles CM, Lee HA (2011). The effect of transdermal testosterone gel pretreatment on controlled ovarian stimulation and IVF outcome in low responders. Fertil Steril.

[21] Bosdou JK, Venetis CA, Kolibianakis EM, Toulis KA, Goulis DG, Zepiridis L, Tarlatzis BC (2012). The use of androgens or androgen-modulating agents in poor responders undergoing in vitro fertilization: a systematic review and meta-analysis. Hum Reprod Update.

[22] Kolibianakis EM, Venetis CA, Diedrich K, Tarlatzis BC, Griesinger G (2009). Addition of growth hormone to gonadotrophins in ovarian stimulation of poor responders treated by in-vitro fertilization: a systematic review and meta-analysis. Hum Reprod Update.

[23] Casson PR, Santoro N, Elkind-Hirsch K, Carson SA, Hornsby PJ, Abraham G, Buster JE (1998). Postmenopausal dehydroepiandrosterone administration increases free insulin-like growth factor-I and decreases high-density lipoprotein: a six-month trial. Fertil Steril.

[24] Weissman A, Horowitz E, Ravhon A, Golan A, Levran D (2011). Dehydroepiandrosterone supplementation increases baseline follicular phase progesterone levels. Gynecol Endocrinol.

[25] Strauss S, Greve T, Ernst E, Fraidakis M, Grudzinskas JG, Andersen CY (2014). Administration of DHEA augments progesterone production in a woman with low ovarian reserve being transplanted with cryopreserved ovarian tissue. J Assist Reprod Genet.

